# Capturing the transition from intermediate to neovascular AMD: Longitudinal changes in choroidal volume and choroidal vascularity index

**DOI:** 10.1007/s00417-025-06767-z

**Published:** 2025-02-13

**Authors:** Enrico Borrelli, Abdul Rasheed Mohammed, Costanza Barresi, Kiran Kumar Vupparaboina, Pasquale Viggiano, Francesco Boscia, Alessandro Berni, Ugo Introini, Michele Reibaldi, Francesco Bandello, Jay Chhablani

**Affiliations:** 1 Department of Surgical Sciences, University of Turin, Turin, Italy; 2 Department of Ophthalmology, City of Health and Science Hospital, Turin, Italy; 3 School of Optometry and Vision Science, University of Waterloo, Waterloo, ON Canada; 4 IRCCS Ospedale San Raffaele Milan, Milan, Italy; 5 Department of Ophthalmology, University Vita-Salute San Raffaele, Milan, Italy; 6 Department of Ophthalmology, University of Pittsburgh School of Medicine, Pittsburgh, PA USA; 7 Department of Translational Biomedicine Neuroscience, University of Bari Aldo Moro, Bari, Italy

**Keywords:** Age-related macular degeneration, Optical coherence tomography, Choroid, Choroidal vascularity index, Anti-VEGF

## Abstract

**Background:**

To perform a three-dimensional assessment of the choroid, including choroidal volume and choroidal vascularity index (CVI), during the transition from intermediate to neovascular age-related macular degeneration (AMD), and following anti-VEGF therapy.

**Methods:**

A total of 42 participants (42 eyes) with intermediate AMD at baseline who developed neovascular AMD within 3 months were included in the analysis. Optical coherence tomography (OCT) scans at follow-up visits (after transition to neovascular AMD and 12 months after the initiation of anti-VEGF therapy) were compared with values at the latest visit with evidence of intermediate AMD to quantify longitudinal choroidal changes. Enhanced depth imaging (EDI) OCT scans were analyzed to obtain the following metrics: (i) choroidal volume, (ii) choroidal stromal volume, (iii) choroidal vascular volume, and (iv) CVI.

**Results:**

At baseline, the mean (median; IQR) choroidal volume (i.e., including both the stromal and vascular components) was 0.156 mm³ (0.149; 0.065), increasing to 0.163 mm³ (0.148; 0.068) at the follow-up when treatment-naïve exudative MNV was first detected (*p* = 0.013). After 12 months of anti-VEGF therapy, it returned to near-baseline levels at 0.156 mm³ (0.146; 0.065; *p* = 0.457). Similarly, the choroidal stromal and choroidal volumes increased at MNV detection but returned to baseline after treatment. Conversely, no alterations in CVI were observed between the baseline and follow-up visits.

**Conclusion:**

The transition from intermediate to exudative neovascular AMD is associated with a significant increase in choroidal volume, affecting both stromal and luminal components. After anti-VEGF treatment, these changes regress, returning to baseline levels.

## Abstract (highlights)

**Key messages:**

*****What is known***:**

- The choroid is affected in patients with AMD.

*****What is new***:**

- The progression from intermediate to exudative neovascular AMD is marked by a notable increase in choroidal volume, impacting both the stromal and luminal regions.
- Following effective anti-VEGF treatment, these changes reverse, returning to baseline levels.

## Introduction

Age-related macular degeneration (AMD) is a prevalent cause of significant central vision loss among elderly individuals in Western countries [1]. While intermediate AMD is typically associated with the presence of large drusen and/or pigmentary alterations, the advanced stage of AMD is distinguished by the emergence of either macular neovascularization (MNV) or geographic atrophy (GA) [2, 3]. The neovascular exudative form of AMD, affecting around 10 to 15% of AMD patients, is characterized by the leakage from pathological type 1 (sub-retinal pigment epithelium - RPE), type 2 (sub-retinal), or type 3 (intra-retinal) MNV [2, 4–8]. 

AMD is primarily considered as a condition resulting from damage to the complex unit consisting of photoreceptors, retinal pigment epithelium (RPE), Bruch’s membrane, and the choroid [9–14]. The choroid represents the most posterior part of the uvea. Histologically, it comprises various structures, including vessels, as well as supportive collagenous and elastic connective tissues, forming the choroidal stroma [15]. Additionally, the choroidal vasculature can be categorized into three sublayers: the choriocapillaris (CC) and Haller’s and Sattler’s layers [15]. 

Recent advancements in imaging technologies have greatly improved our ability to study and characterize the choroid [16]. Advancements such as enhanced depth imaging (EDI) and swept source technologies have significantly ameliorated the ability of structural optical coherence tomography (OCT) to provide morphological information about the choroid and to assess medium- and larger-sized choroidal vessels and stroma [17]. Although previous studies have primarily focused on choroidal thickness to investigate the choroid, this measure alone does not provide insights into the vascular and stromal components. The choroidal vascular index (CVI) is a parameter derived from structural OCT images, calculated by determining the proportion of the luminal component to the entire choroid, which encompasses both luminal and stromal components. It is worth noting that both luminal and stromal component, and CVI parameters have been shown to be influenced by various physiological and pathological conditions including AMD [18–29], given that the choroid is known to be significantly affected in the latter disease [16, 30, 31]. More importantly, the choroid seems to play a clinically significant role in AMD, as notable changes in this vascular layer have been linked to disease progression and, in the case of neovascular AMD, to disease recurrence [28, 32–34]. However, no study has yet examined the choroid during the transition between different stages of the disease.

The current study examined the longitudinal three-dimensional volumetric changes in the choroid following the transition from intermediate to exudative neovascular AMD. Additionally, we assessed the same variables at the 12-month follow-up visit after the resolution of exudation following anti-VEGF therapy. Importantly, we conducted association analyses between choroidal metrics and the presence and distribution of exudation in these eyes.

## Methods

The San Raffaele Ethics Committee was informed of this retrospective cohort study, which adhered to the 1964 Helsinki Declaration and its subsequent revisions. A waiver of informed consent was obtained to analyze previously collected data.

### Subjects

In this study, we analyzed subjects with AMD who were part of a study cohort previously examined in another study [35]. In the latter study, 80 patients (80 eyes) were analyzed. Beyond the criteria used in the previous study, patients were also required to have OCT images acquired with a pattern of six radial linear B-scans using enhanced depth imaging (EDI), centered on the fovea. Additionally, all OCT B-scans were required to clearly capture the entire choroid. These requirements led to the exclusion of 38 patients, resulting in a final cohort of 42 patients (42 eyes) for the present study [35]. 

In detail, we identified consecutive subjects aged 50 years and older with a diagnosis of intermediate AMD in at least one eye from the San Raffaele Scientific Institute. To be eligible for inclusion, subjects were required to develop treatment-naïve exudative neovascular AMD within 3 months after their latest visit, demonstrating evidence of intermediate AMD. Diagnoses of intermediate and exudative neovascular AMD were confirmed through clinical examination, structural OCT, and dye angiography, when necessary, as previously outlined [3, 36]. Following the diagnosis of exudative neovascular AMD, all patients received an initial loading dose of anti-VEGF intravitreal injections, followed by either a pro re nata (PRN) or treat-and-extend regimen at the discretion of the treating physician. Ultimately, included eyes needed to show evidence of resolved exudation 12 ± 1 months after the initiation of anti-VEGF therapy to prevent exudation from affecting the 12-month OCT quantitative and qualitative analysis. The exclusion criteria included: (i) previous vitreoretinal surgery; (ii) any maculopathy not related to AMD; (iii) intraocular pressure exceeding 20 mmHg; (iv) presence of diabetic retinopathy or any optic neuropathy.

OCT imaging was performed in the morning, a factor that is important given the diurnal variation in choroidal volume [37, 38]. Each B-scan consisted of 25 averaged OCT images. A minimum signal strength of 25 was necessary for the OCT images to be included, as per the manufacturer’s recommendation [39]. It was essential that OCT images from both baseline and follow-up examinations clearly displayed the entire choroid in all 6 radial linear B-scans. The final cohort analyzed comprised 42 subjects.

The analysis included the following visits: (i) baseline assessment (i.e., 0 to 3 months before the detection of treatment-naïve exudative neovascular AMD); (ii) follow-up visit with the first evidence of treatment-naïve exudative MNV; and (iii) the 12-month follow-up visit after the initiation of anti-VEGF therapy. As mentioned above, the latter visit was characterized by absence of signs of exudation.

During each visit, a comprehensive ophthalmologic examination was conducted. Structural OCT images were acquired using the Heidelberg Spectralis HRA + OCT device (Heidelberg Engineering, Heidelberg, Germany). In addition to the scan comprising 6 radial linear B-scans, volumetric scans centered on the fovea, consisting of 19 horizontal B-scans, were also conducted.

## OCT analysis

**Fig. 1:**
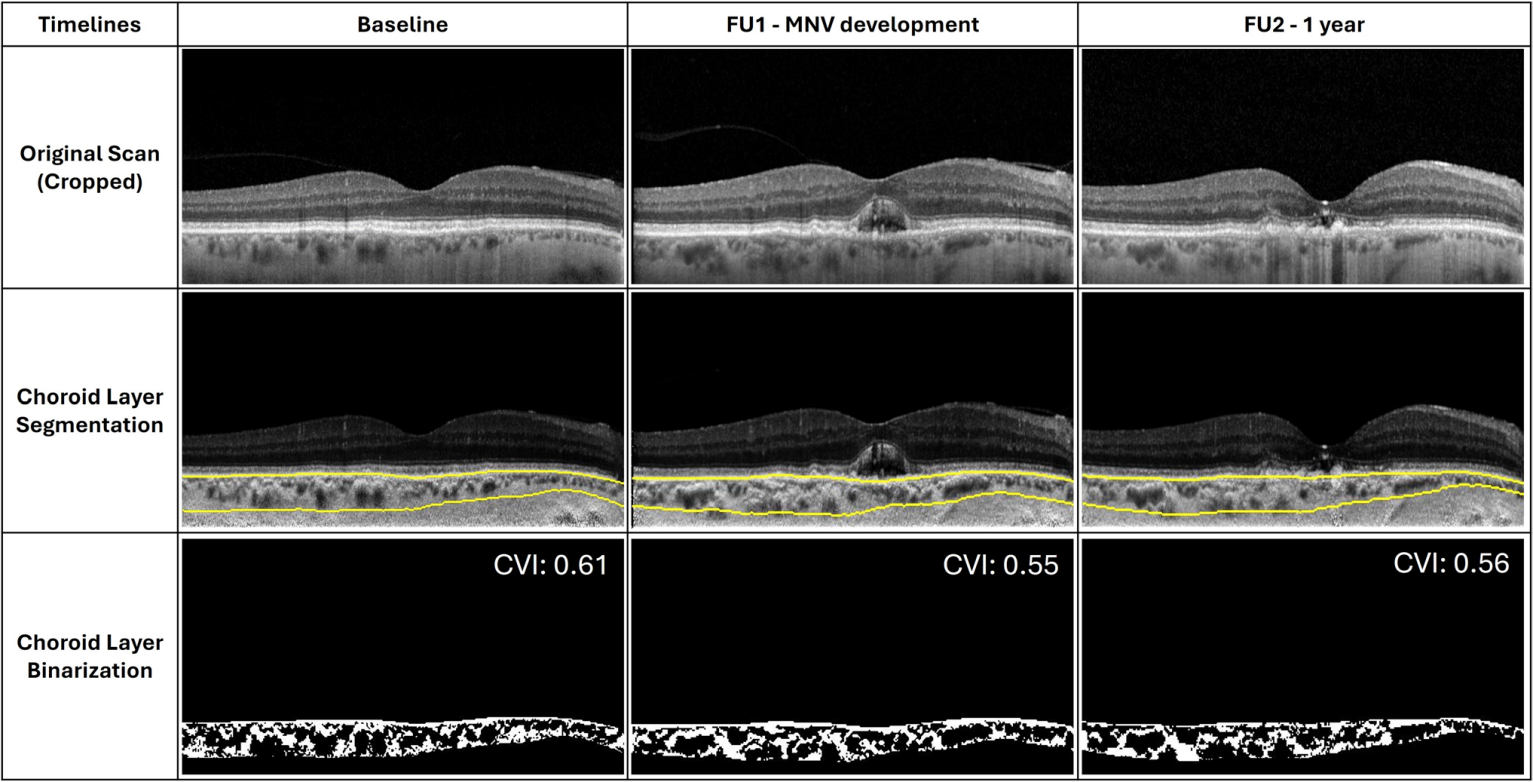
Representative optical coherence tomography (OCT) B-scans of a patient transitioning from intermediate to neovascular AMD. At the initial assessment (left column), the patient displays signs of intermediate AMD. In the subsequent evaluation (middle column), there is the first indication of treatment-naïve neovascular AMD. After receiving anti-VEGF injections, the patient showed no signs of exudation at the 12-month follow-up (right column). The top row presents the original OCT B-scans, the middle row highlights the same images with yellow boundaries indicating the segmented choroid, and the bottom row illustrates the binarization of the choroidal area

Three-dimensional choroidal parameters were assessed using a pre-established automated algorithm, as detailed in prior publications (Fig. 1) [40–42]. This algorithm involved several steps, including shadow compensation, denoising, identification of the inner and outer choroidal boundaries, and segmentation of the choroidal region from the remainder of the scan. The choroid was then binarized, resulting in bright regions representing stromal choroidal areas and dark regions representing luminal choroidal areas. The latter analysis was made to calculate the choroidal stromal volume and choroidal vascular volume metrics, which represent imaging surrogate of stromal and luminal volumes, respectively. The total choroidal volume was obtained by summing these two metrics. Finally, CVI was calculated as the ratio of the choroidal vascular volume and total choroidal volume. These measurements were conducted at the following visits: (i) baseline visit (i.e., the most recent visit before the transition from intermediate to exudative neovascular AMD); (ii) follow-up visit with first evidence of treatment-naïve exudative MNV; and (iii) the 12-month follow-up visit after the initiation of anti-VEGF therapy, when a complete resolution of signs of exudation was obtained.

In addition, two readers (EB and CB) independently assessed eligible eyes for qualitative OCT features indicative of MNV exudation, including presence of intraretinal fluid (IRF), subretinal fluid (SRF), and subretinal hyperreflective material (SHRM), as outlined previously [43]. This grading was carried out at the only visit that could be characterized by exudation as per study design (i.e., follow-up visit with first evidence of treatment-naïve exudative MNV).

### Statistical analysis

Statistical calculations were performed using Statistical Package for Social Sciences (version 20.0, SPSS Inc., Chicago, IL, USA). To detect normal distribution, a Shapiro-Wilk’s test was performed for all variables. Visual acuities and OCT metrics at baseline and follow-up visits were compared by conducting paired-samples T test or related-samples Wilcoxon signed rank test. Contributory factors affecting percentage change in choroidal volume (dependent variable) were examined using univariable linear regression analysis [32, 33]. 

## Results

### Characteristics of patients included in the analysis

Forty-two eyes from 42 patients with AMD (11 males and 31 females) were included in this study. The average age at baseline was 79.1 ± 7.7 years. A summary of the demographics and clinical characteristics of the study group is provided in Table 1. The average time between the baseline visit and the first visit showing evidence of macular neovascularization (MNV) was 74.7 ± 21.4 days. Median visual acuity (IQR) was 0.10 (0.14) LogMAR at the baseline assessment (i.e., the most recent visit showing evidence of intermediate AMD, occurring 0 to 90 days prior to detection of treatment-naïve neovascularization), 0.20 (0.20) LogMAR at the follow-up visit where treatment-naïve exudative MNV was first detected, and 0.20 (0.40) LogMAR at the 12-month follow-up visit after starting anti-VEGF therapy (*P* < 0.001 for all comparisons with baseline values). Of the 42 eyes, 22 (52.4%) developed type 1 MNV, while 9 (21.4%) and 11 (26.2%) eyes developed type 2 and type 3 MNV, respectively. At the follow-up visit where treatment-naïve exudative MNV was first observed, 27 eyes (64.3%) had OCT evidence of SHRM, 34 eyes (81.0%) were graded to be affected by SRF, and 20 eyes (47.6%) had IRF.

**Table 1:** Demographic and clinical characteristics of enrolled patients

Number of patients, *n*	42
Number of eyes, *n*	42
Age (years), mean (SD)	79.1 (7.7)
Gender, *n*	
Male	11
Female	31
Number of anti-VEGF injections in the study period, mean (SD)	6.1 (2.7)

*n* number, *SD* standard deviation, *VEGF* vascular endothelial growth factor

### Longitudinal changes in choroidal metrics

The mean (median; IQR) choroidal volume at baseline was 0.156 mm [3] (0.149; 0.065), which increased to 0.163 mm [3] (0.148; 0.068) at the follow-up visit when treatment-naïve exudative MNV was first detected (*p* = 0.013). However, after 12 months of anti-VEGF therapy, the choroidal volume numerically decreased with a mean (median; IQR) of 0.156 mm [3] (0.146; 0.065; *p* = 0.457 vs. baseline) (Table 2). Consistently, the choroidal stromal volume was 0.061 mm [3] (0.056; 0.028) at baseline and increased to 0.064 mm [3] (0.061; 0.029) (*p* = 0.040) when MNV was first detected. After anti-VEGF therapy, values returned not significantly different as compared with baseline with a mean (median; IQR) value of 0.061 mm [3] (0.058; 0.025) (*p* = 0.807) (Table 2). Similarly, the choroidal vascular volume was different between baseline and follow-up visit with first evidence of MNV [baseline: 0.093 mm [3] (0.090; 0.037); follow-up: 0.097 mm [3] (0.086; 0.046)] (*p* = 0.047). Similarly to the other metrics, the choroidal vascular volume returned to baseline values after anti-VEGF therapy with mean (median; IQR) values of 0.092 mm [3] (0.084; 0.039) (*p* = 0.303) (Table 2). Conversely, no alterations in CVI were observed between the baseline and follow-up visits (Table 2).

**Table 2:** Choroidal parameters at different visits

	Baseline visit (latest evidence of intermediate AMD)	Follow-up visit with first evidence of treatment-naïve exudative MNV	12-month follow-up visit following the initiation of anti-VEGF therapy
Choroidal volume (mm^3^), mean; median (IQR)	0.156; 0.149 (0.065)	0.163; 0.148 (0.068)	0.156; 0.146 (0.065)
		0.013^a^	0.457^a^
Choroidal stromal volume (mm^3^), mean; median (IQR)	0.061; 0.056 (0.028)	0.064; 0.061 (0.029)	0.061; 0.058 (0.025)
		0.040^a^	0.807^a^
Choroidal vascular volume (mm^3^), mean; median (IQR)	0.093; 0.090 (0.037)	0.097; 0.086 (0.046)	0.092; 0.084 (0.039)
		0.047^a^	0.303^a^
Choroidal vascularity index, mean; median (IQR)	0.598; 0.596 (0.031)	0.595; 0.591 (0.023)	0.590; 0.591 (0.025)
		0.549^a^	0.220^a^

*AMD* age-related macular degeneration, *MNV* macular neovascularization, *VEGF* vascular endothelial growth factor, *IQR* interquartile range

^a ^*p* value comparison versus baseline visit

The percentage change in choroidal volume between the baseline visit, prior to the transition to neovascular AMD, and the follow-up visit when treatment-naïve exudative MNV was first detected, was 4.5 ± 11.2%. In the univariable regression analysis, none of the demographic or clinical factors included as independent variables were associated with this percentage change (Table 3).

**Table 3:** Results of univariable analysis at the follow-up visit with first evidence of treatment-naïve exudative MNV and percentage change in choroidal volume as dependent variable

	Standardized β Coefficient (95 % CI B)	*P* value
Independent variables		
Age	0.00229 (−0.00494, 0.00434)	0.897
MNV type	0.0206 (−0.0381, 0.0452)	0.864
IRF: Y/N	0.0346 (−0.0388, 0.1011)	0.374
SRF: Y/N	0.0426 (−0.1661, 0.00619)	0.068
SHRM: Y/N	0.0352 (−0.0117, 0.1305)	0.099

*SE* standard error, *Y* yes, *N* no, *MNV* macular neovascularization, *IRF* intraretinal fluid, *SRF* subretinal fluid, *SHRM* subretinal hyperreflective material

## Discussion

In this longitudinal study, we provide a three-dimensional quantitative analysis of the choroid in patients with AMD complicated by exudative MNV who were treated with anti-VEGF therapy. Overall, we found that eyes with AMD exhibit longitudinal changes in the choroid. Notably, our findings revealed a significant increase in choroidal volume at the onset of exudative neovascularization, which regressed one year after the initiation of anti-VEGF therapy.

As mentioned above, binarization of OCT images to examine changes in the stromal and vascular components of the choroid, as well as the CVI, has been widely used in patients with AMD [24, 25, 28, 29], as the choroid is known to be significantly affected in these individuals [16, 30, 31]. In an important study, Koh and colleagues [28] analyzed EDI-OCT scans from 64 patients with unilateral or bilateral AMD and compared choroidal metrics with those of 18 age-matched healthy controls. The study found that the choroid exhibited shrinkage in AMD patients, with a lower CVI, this suggesting that the vascular component is more affected in these patients, while the stromal component that may be less impacted. The CVI is a parameter that may reflect an imbalance between changes in the vascular and stromal components of the choroid [19]. In another study, Sacconi et al. [24] utilized these choroidal metrics obtained using structural OCT to compare various cohorts of individuals with dry AMD and healthy subjects. In particular, the study enrolled 120 eyes of 120 Caucasian patients, who were classified into four sub-groups based on AMD classification: those with only drusen, individuals with reticular pseudodrusen (RPD), patients with geographic atrophy, and healthy controls. The latter study highlighted a shrinking of the choroid among patients with AMD, particularly notable in eyes with GA and RPD, as compared with eyes with only drusen. Interestingly, also in this study, the reduction was more prominent in the luminal part of the choroid rather than its stromal component. In another study, Invernizzi and colleagues [25] conducted a retrospective analysis of AMD eyes that underwent two sequential visits within a 12-month period and exhibited either no MNV or inactive MNV at the initial visit. The researchers examined choroidal thickness and CVI using horizontal OCT B-scans passing through the fovea, which limited the assessment to a two-dimensional analysis. Their findings indicated a significant increase in choroidal thickness and CVI during active disease in eyes with neovascular AMD. Another limitation of the study by Invernizzi et al. [25] was that not all patients had intermediate AMD before progressing to exudative neovascular AMD, as some patients had neovascular AMD without any signs of exudation at the first assessment. In another study, Boscia and colleagues [29] investigated choroidal changes in 73 eyes with treatment-naïve exudative type 1 MNV at baseline to understand the effects of a loading dose of anti-VEGF therapy on the choroid. The authors found a significant reduction in choroidal area after anti-VEGF treatment, along with a notable decrease in signs of exudation.

As mentioned above, exudative neovascular AMD may be associated with choroidal enlargement [25], which eventually tends to decrease after anti-VEGF therapy [29]. However, previous studies did not examine the transition from intermediate to neovascular AMD and did not evaluate changes after the resolution of exudation in all patients. Additionally, these earlier reports were limited to a two-dimensional assessment. Some of the earlier reports performed CVI calculations without shadow compensation, thus impact of retinal tissue on choroidal analysis wouldn’t provide accurate assessment [44]. Many of the earlier studies have used single B scan analysis, which is yet again another weakness, as the volumetric assessment of choroid is essential in AMD instead of single B scan. In contrast, the present longitudinal study addressed this limitation by capturing the transition from intermediate to exudative neovascular AMD and performing a volumetric longitudinal assessment of the choroid in a cohort of AMD patients.

In our study, we observed a significant increase in choroidal volume at the follow-up visit where treatment-naïve neovascular AMD was first detected. However, after one year of follow-up, following the onset of exudative MNV, the choroidal volume returned to levels similar to those observed at baseline, before the development of exudative neovascular AMD. Notably, we conducted a more detailed longitudinal assessment by dividing the volumetric changes into the stromal and luminal (i.e., vascular) components of the choroid. Specifically, the choroidal stromal volume and choroidal vascular volume metrics were used to evaluate the stromal and vascular components, respectively. This analysis revealed that the observed longitudinal changes in choroidal volume affected both components equally, indicating that the changes in choroidal volume involved both the stromal and vascular elements of the choroid without distinction. Consequently, we did not observe any change in the CVI throughout the follow-up period, as this metric represents the ratio of the luminal component to the entire choroid. Assuming that both stromal and vascular components change accordingly, the CVI remained unchanged throughout the follow-up period.

The increase in choroidal volume following the development of exudative MNV may be associated with different factors. In AMD eyes, retinal and RPE cells are known to release VEGF, which dilates choroidal vessels and increases blood flow [28, 45–47]. This rise in VEGF levels may also promote the development and exudation of MNV [9, 13]. Consequently, choroidal thickening may occur in AMD eyes when neovascularization develops and starts to exudate, driven by elevated VEGF levels and other proinflammatory factors. However, in our study, we observed that the volume increase affected both the stromal and luminal components of the choroid, suggesting that VEGF-induced vasodilation may not be the only mechanism involved. Another potential explanation for the increased choroidal volume at the follow-up visit when treatment-naïve neovascular AMD was first detected, could be MNV leakage within the choroidal space. However, this alone does not fully account for our findings, as no associations were observed between OCT signs of exudation and changes in choroidal volume. Therefore, we propose that both mechanisms may contribute to the increase in choroidal volume observed during the transition from intermediate to exudative neovascular AMD. Regardless of the underlying mechanism, after one year of treatment, when MNV exudation is resolved and VEGF levels are reduced, we observed a return to baseline choroidal volume values. An important finding in our study was that the results were independent of the type of neovascularization. While type 1 and type 2 MNV originate from the choroid, with exudation primarily occurring within the choroid, type 3 MNV originates from the deep vascular complex of the retinal vasculature [37, 38, 48]. However, after originating in the deep vascular complex, type 3 MNV extends deeper into the retina, reaching the RPE and resulting in exudation within the sub-RPE space [49]. Therefore, although type 3 MNV originates from the retinal vasculature, it is not surprising that its exudation may contribute to an increase in choroidal volume.

Our study does have limitations, including its retrospective design, which may be prone to selection and ascertainment bias. However, designing a prospective study would be challenging, as predicting the exact timing of neovascular AMD development is not feasible. Additionally, our study cohort consisted of subjects with resolved exudation at the 12-month follow-up after initiating anti-VEGF therapy, so our findings may not be applicable to all cases. Nonetheless, our primary goal was to understand how the choroid changes once signs of exudation have resolved. Therefore, we decided it was safer to exclude cases with OCT evidence of exudation at the final follow-up visit. Moreover, we could not assess fellow eyes in patients with unilateral disease, as only 3 subjects had unilateral disease with complete imaging to enable such analysis. Since we were not able to include eyes with intermediate AMD that did not progress to neovascular AMD, we could not rule out the possibility that these changes were part of the natural progression of AMD rather than the transition itself.

In conclusion, this study examined volumetric longitudinal changes in the choroid following the transition from intermediate to exudative neovascular AMD. We found that the development of exudative neovascularization is associated to a significant increase in choroidal volume, affecting both the stromal and luminal components. Notably, we observed a return to pre-transition choroidal values once exudation had resolved. These findings may enhance our understanding of the pathogenesis and natural history of AMD. Importantly, these results should be considered in both research and clinical practice, given the widespread use of quantitative choroidal measurements in these patients. If replicated in larger future studies, choroidal volume could potentially serve as a biomarker associated with exudation in AMD patients.
